# IgG Avidity in Samples Collected on Filter Paper: Importance of The Early Diagnosis of Congenital Toxoplasmosis

**DOI:** 10.1055/s-0041-1740272

**Published:** 2021-12-21

**Authors:** Jéssica Yonara de Souza, Taynara Cristina Gomes, Hanstter Hallison Alves Rezende, Heloisa Ribeiro Storchilo, Patrícia Giffron Rodrigues, Ana Maria de Castro

**Affiliations:** 1 Universidade Federal de Goiás, Goiânia, GO, Brazil

**Keywords:** toxoplasmosis, IgG avidity, filter paper, neonatal screening, pregnancy, toxoplasmose, avidez de IgG, papel filtro, triagem neonatal, gravidez

## Abstract

**Objective** The purpose of the present study is to standardize and evaluate the use of the immunoglobulin G (IgG) antibody avidity test on blood samples from newborns collected on filter paper to perform the heel test aiming at its implementation in ongoing programs.

**Methods** Blood samples from newborns were collected on filter paper simultaneously with the heel prick test. All samples were subjected to immunoglobulin M IgM and IgG enzyme-linked immunosorbent assays (ELISA). Peripheral blood was collected again in the traditional way and on filter paper from newborns with high IgG levels ( ^3^ 3). Three types of techniques were performed, the standard for measuring IgG in serum, adapted for filter paper and the technique of IgG avidity in serum and on filter paper. The results of the avidity test were classified according to the Rahbari protocol.

**Results** Among the 177 samples, 17 were collected in duplicate from the same child, 1 of peripheral blood and 1 on filter paper. In this analysis, 1 (5.88%) of the 17 samples collected in duplicate also exhibited low IgG avidity, suggesting congenital infection. In addition, the results obtained from serum and filter paper were in agreement, that is, 16 (94.12%) samples presented high avidity, with 100% agreement between the results obtained from serum and from filter paper.

**Conclusion** The results of the present study indicate that the avidity test may be another valuable method for the diagnosis of congenital toxoplasmosis in newborns.

## Resumo

**Objetivo** O objetivo do presente estudo é padronizar e avaliar a utilização do teste de avidez de anticorpos imunoglobulina G (IgG) em amostras de sangue de recém-nascidos (RNs) coletadas em papel filtro para a realização do teste do pezinho visando a implementação nos programas já vigentes.

**Métodos** Foram coletadas amostras de sangue de recém-nascidos em papel filtro simultaneamente ao teste do pezinho. Em todas as amostras, foram realizados os testes imunoenzimáticos (ELISA) imunoglobulina M (IgM) e IgG. Dos RNs que apresentaram altos índices de IgG ( ^3^ 3), foi novamente coletado sangue periférico da forma tradicional e em papel filtro. Foram realizadas técnicas padrão para a dosagem de IgG em soro, adaptadas para papel filtro, e a técnica de avidez de IgG em soro e em papel filtro. Os valores obtidos para o teste de avidez foram classificados de acordo com o protocolo de Rahbari.

**Resultados** Dentre as 177 recoletas, em 17 amostras foi realizada a coleta simultânea de sangue periférico e papel filtro da mesma criança. Nesta análise, 1 (5,88%) das 17 amostras coletadas em duplicata obteve também baixa avidez de IgG, sugerindo infecção congênita da criança, e houve concordância entre os resultados obtidos em soro e em papel filtro: 16 (94,12%) das amostras apresentaram alta avidez, com concordância de 100% entre os resultados obtidos em soro e em papel filtro.

**Conclusão** Os dados do presente trabalho evidenciam que o teste de avidez poderá ser mais um método valioso a ser utilizado no diagnóstico da toxoplasmose congênita em RNs.

## Introduction

Congenital toxoplasmosis is an infectious disease caused by the transplacental transfer of *Toxoplasma gondii* tachyzoites from the primary infection of the mother, by reinfection, or by resurgence of a previous infection, and is particularly relevant because of the damage inflicted on the developing fetus. 1

Toxoplasmosis is one of the most harmful diseases for the fetus, particularly when the mother becomes infected in the 1 ^st^ 2 trimesters of pregnancy. 2 Studies in Brazil have revealed a prevalence of congenital toxoplasmosis of between 3 and 20 per 10,000 live births. 3 4 5 6 Approximately 80% of vertically infected children show no symptoms at birth, but later exhibit signs of the disease, mainly with ocular, motor, and central nervous system involvement. 7 8

The importance of diagnosing active infection by *T. gondii* during pregnancy and of confirming congenital transmission in newborns (NBs) cannot be overstated, because it allows for the adoption of measures of primary and secondary care, minimizing serious impairments caused by congenital transmission. 9

In the routine laboratory tests offered by the Brazilian Unified Health System (SUS, in the Portuguese acronym), toxoplasmosis is diagnosed by means of serological tests based on the detection of specific antibodies of the classes of immunoglobulin M (IgM) and immunoglobulin G (IgG), mainly by means of the enzyme-linked immunosorbent assay (ELISA) method. 10 However, assistance provided to pregnant women during prenatal care is still not satisfactory. Pregnant women often have access to exams only in the last month of pregnancy, when prenatal tests are performed in programs that use filter paper for serological screening. This situation is one of the main factors that limit the control and the prevention of infection, of confirmation of risk, and of congenital transmission. 11

The diagnosis of toxoplasmosis is complex, and monitoring the NBs of mothers infected with *T. gondii* , confirming the infection, and providing early treatment, are crucial for the prognosis of the newborn. 12 The literature recommends the IgG avidity test because it is a fast and inexpensive technique, and is an auxiliary method for optimization of the diagnosis of recent infection, and, therefore, of congenital toxoplasmosis. This recommendation favors the implementation of this technique in public health programs, especially in Brazil, where the incidence of congenital toxoplasmosis is high.

The infection is usually confirmed by laboratory tests that identify the parasite or by the presence of specific antibodies that do not cross the placental barrier (IgA, IgM or IgE) in the blood of the patient. 13 Immunoglobulin G, which is a marker of chronic infection and crosses the transplacental barrier, is still not used as a marker of congenital infection. However, when IgG levels in NBs differ from maternal levels, they may suggest infection. 14

The functional affinity of IgG antibodies for antigens is low in the primary antigen response and increases when the immune system reaches maturity. 15 The IgG antibody avidity test analyzes the binding affinity of the antigen-antibody (AG-AB) complex. The AG-AB bond is easily dissociated in the acute phase of the disease because the synthesis of antibodies is recent. This is why IgG has low avidity for antigens, which are considered to be of low avidity (< 30%, depending on what kits and protocols are used). On the other hand, AG-AB complexes are difficult to dissociate in the chronic phase, exhibiting high IgG avidity, that is, late synthesis of antibodies considered to have high avidity. 16

Therefore, the purpose of the present study was to standardize and assess the use of the IgG antibody avidity test on blood samples from NBs in order to optimize it and employ it in existing programs that use filter paper to collect blood for the neonatal heel prick test.

## Methods

The present research project was submitted to the Brazilian online system for human subject research proposals, Plataforma Brasil, and was accepted under the Protocol No. 943,441 on February 11, 2015. Blood samples were collected between April 2016 and February 2017 at the maternity wards of the Hospital das Clínicas da Universidade Federal de Goiás (HC-UFG, in the Portuguese acronym) and of the Hospital e Maternidade Dona Iris (HDMI, in the Portuguese acronym), both in the municipality of Goiânia, state of Goiás, Brazil, and at the maternity ward of the Cais Nova Era (CNE, in the Portuguese acronym) in Aparecida de Goiânia, state of Goiás, Brazil. These maternity wards were chosen for convenience, and the patients came from the public health network of Goiânia, state of Goiás, Brazil.

The samples were collected on filter paper at the same time as the heel prick test was applied, upon the consent of the person responsible for the NB. The blood was collected from the lateral plantar region of the NB on Wathman Grade 1 filter paper, with the NB held in the burping position to ensure good blood circulation in the feet. After the circles marked on the filter paper were filled with blood, the papers were tagged and placed on horizontal shelves to dry for ∼ 3 hours at room temperature, between 15 and 20°C, avoiding contact with other samples. The dry biological samples were then sent to the Laboratory for Parasite-Host Relationship Studies at the Universidade Federal de Goiás (LAERPH-UFG, in the Portuguese acronym).

All samples were subjected to IgM and IgG ELISA using Quibasa-Bioclin kits.

Newborns presenting higher IgG levels than the average level of patients tested in the LAERPH routine, that is, patients with a level ≥ 3, served as a risk criterion for congenital infection. A new peripheral blood sample was collected from these infants, drawn in the traditional manner and on filter paper, within a period of up to 3 months after the 1 ^st^ blood sample collection, for confirmation and comparison of the serology on filter paper and for the IgG avidity test of the infant.

Avidity was evaluated using the protocol of Rahbari et al., 17 with some adaptations for filter paper, as described in detail in Chart 1 .

**Chart 1:** ELISA technique (immunoenzymatic assay) according to the protocol of the manufacturer of the Quibasa-Bioclin kit, a technique adapted for IgG avidity, and standardized in house with changes for the use of samples collected on filter paper

Standard technique to measure serum IgG levels	Technique adapted to measure IgG levels on filter paper	Technique to determine IgG avidity in serum and on filter paper
100 μl of sample diluent + 5 μl of sample	Perforation with specific 5mm diameter perforator + 10 μl of sample diluent	Serum: 100 μl of sample diluent + 5 μl of sample Filter paper: 100 μl of sample diluent + one 5mm diameter filter paper disc
Incubation for 30 minutes at 37°C	Incubation for 30 minutes at 37°C	Incubation for 30 minutes at 37°C
First wash with 300 μl of washing solution previously prepared according to the manufacturer's instructions	Removal of filter papers and first wash with 300 μl of washing solution previously prepared according to the manufacturer's instructions	First wash with 300 μl of previously prepared solution of 6M Urea
Addition of 100 μl of conjugate		
Incubation for 30 minutes at 37°C		
Second wash with 300 μl of washing solution previously prepared according to the manufacturer's instructions		
Addition of 50 μl of substrate A and 50 μl of substrate B		
Incubation for 10 minutes at 37°C		
Addition of 50 μl of stop solution		
Reading on filters at 450nm and 630nm		

Abbreviations: IgG, Immunoglobulin G.

**Source:** Rahbari et al. 17

Two plates, A and B, were used simultaneously to determine the reactivity. The serum samples were diluted 1:200 and 100 μl were added per well. After incubation, plate A was washed 5 times with sample buffer from the Bioclin commercial kits (Quibasa Química Básica Ltda), while plate B was washed 3 times with sample buffer containing 6 Molar urea. The plates were then treated according to the instructions of the manufacturer. To validate the technique and standardize the use of 5-mm filter paper discs, tests were carried out concomitantly using the samples collected on filter paper using the Virion Serion commercial kit for filter paper and the Quibasa-Bioclin commercial kits used in our study. After the readings and technical validations, the cutoff of each plate and the indices of each sample were calculated to determine IgG and IgM. The percentage of IgG avidity was calculated based on the following formula: AI = Abs (U + ) / Abs (U-) x 100, 1 where the result of absorbance of wells washed with PBS-urea (U + ) was divided by the absorbances of wells washed with PBS (U-) and then multiplied by 100. 17 The values obtained in the avidity test were classified according to the protocol proposed by Rahbari et al., 17 and patients with avidity values ≤ 30% were considered as having low IgG avidity.

## Results

A total of 1,277 whole blood samples were collected on filter paper from 3- to 7-day-old NBs in the maternity wards of the HC-UFG, the HMDI, and the CNE. The ELISA test detected the presence of anti- *T. gondii* IgG antibodies in 44.4% (567/1,277) of the analyzed samples. Of the 567 blood samples collected on filter paper that were reactive to IgG, 57.67% (327/567) presented an absorbance value ≥ 3.0 and were considered at risk in the present study. Following the proposed methodology, the mothers of infants whose blood samples on filter paper showed ELISA titers ≥ 3 were contacted and peripheral blood samples were collected from their babies before they were 3 months old ( Table 1 ).

**Table 1:** Comparison of Immunoglobulin G anti- *Toxoplasma gondi* *i* , obtained by the ELISA test on serum samples from children and their respective mothers collected 3 months after birth

Results	Mother		Child	
	Absolute number	%	Absolute number	%
Reagent (IgM)	0	0	0	0
Reagent (IgG)	167	94.36	167	94.36
Reactive mother and non-reactive child (IgG)	5	2.82	5	2.82
Non-reactive mother and child (IgG)	5	2,82	5	2,82
Total samples	177	100	177	100

Abbreviations: IgG, immunoglobulin G; IgM, immunoglobulin M.

1. Comparison of the results of blood on filter paper and peripheral blood

A total of 177 pairs of samples were collected, and the results of 167 (94.36%) peripheral blood samples from NBs were in agreement with those obtained in the heel prick test on filter paper. However, 10 (5.64%) peripheral blood samples showed results that were inconsistent with those obtained on filter paper.

2. Comparison of serology in samples collected simultaneously of peripheral blood of infants and their mothers

All the 177 pairs of recollections (mothers and children) performed showed negative results for IgM. Regarding IgG, 167 pairs (94,36%) of samples from both mother and child were detected with the presence of anti-T *gondii* IgG antibodies.

3. High IgG avidity – filter paper versus peripheral blood

The 167 samples from babies that remained IgG-positive after 3 months were subjected to the IgG avidity test to determine the binding strength of this immunoglobulin with the epitope, in order to ascertain if the infection was a recent or past infection of the mother. Among these 167 samples, 163 (97.60%) showed high avidity of antibodies, and 4 (2.40%) of the samples showed low avidity of IgG antibodies, which is indicative of a recent infection. Congenital infection was then confirmed in 50% of the samples from infants with low IgG antibody avidity by the Western Blot method. For infants with high IgG avidity, the finding must be confirmed by other methods. Among the 167 samples collected in duplicate, 17 samples were randomly collected from peripheral blood and filter paper simultaneously from the same infant. In this analysis, 1 (5.88%) of the 17 samples collected in duplicate also showed low IgG avidity, suggesting congenital infection of the infant, and the results obtained in serum and in filter paper were in agreement. Sixteen (94.12%) of the samples showed high avidity, with 100% agreement between the results obtained in serum and in filter paper, as shown in Table 2 . 17

**Table 2:** Comparison of anti- *Toxoplasma gondii* IgG avidity values obtained by ELISA using Quibasa-Bioclin kits adapted for IgG avidity in serum samples and adapted in house for samples in filter paper, collected simultaneously 3 months after birth

Samples	IgG avidity serum	IgG avidity filter paper	IgG serum (ELISA index)	IgG filter paper (ELISA index)
Patient 1	26%	26%	3.52	3.31
Patient2	52%	56%	2.94	2.75
Patient3	48%	43%	2.65	2.37
Patient4	89%	89%	2.04	1.47
Patient5	61%	87%	1.51	1.71
Patient6	56%	73%	2.0	2.02
Patient7	87%	86%	1.27	0.99
Patient8	95%	77%	16.70	17.21
Patient9	76%	84%	1.24	1.56
Patient10	77%	88%	3.72	2.59
Patient11	81%	91%	5.13	4.60
Patient12	86%	73%	3.44	4.53
Patient13	52%	55%	3.86	3.54
Patient14	72%	81%	2.25	2.18
Patient15	94%	92%	1.88	1.83
Patient16	53%	99%	4.72	2.17
Patient17	52%	55%	3.86	3.54

Abbreviations: ELISA, enzyme-linked immunosorbent assay; IgG, Immunoglobulin G.

**Source:** Rahbari et al. 17

## Discussion

The clinical diagnosis of toxoplasmosis is complex, and sometimes inaccurate, as most pregnant women are asymptomatic. Moreover, when they do present symptoms, these may be mistaken for other infectious agents, such as Cytomegalovirus, *Herpes simplex virus* (HSV-1 or HSV-2), *Rubella virus* , HIV, Epstein Barr, *Treponema pallidum* , *Listeria monocytogenes* , *Borrelia burgdorferi* (Lyme disease), and *Trypanosoma cruzi* (Chagas disease). 18

According to data presented by the Association of Parents and Friends of Disabled Children (APAE, in the Portuguese acronym) in Goiânia, state of Goiás, Brazil, from 2003 to 2013, 9,247,974 prenatal screening tests were performed on mothers in their 1 ^st^ trimester of pregnancy, but only 653,562 pregnant women underwent prenatal tests in the 3 ^rd^ trimester. In other words, ∼ 93% of mothers did not undergo the recommended toxoplasmosis screening test during pregnancy, although this is the third most frequent disease diagnosed among the 24 tests performed during prenatal care on a total of 27,924 pregnant women with confirmed infections, while hepatitis B and syphilis rank in first and second place, respectively. 19

Neonatal infection is usually asymptomatic, and when identified, may present clinical signs similar to erythroblastosis fetalis and to certain degenerative diseases. The clinical examination only suggests the possibility of this etiology, even when toxoplasmosis is symptomatic. 20

Most studies, including those by Buffolano et al. 21 and Cañedo-Solares et al., 22 focus on the predictive value of the IgG avidity test in pregnant women, demonstrating the importance of this test in the diagnosis during the acute phase of the infection, with 100% sensitivity and 92.7% specificity. 23 24 However, few studies in the literature focused on the use of avidity in the blood of NBs, and observed low IgG avidity values in infected NBs. 25

The focus of the present study was to measure and analyze the levels of IgG and test the IgG avidity in peripheral blood from NBs, following the aforementioned criteria. However, based on our findings and on the promising potential of this method in the early diagnosis of congenital toxoplasmosis, the possibility of validating an IgG avidity technique on filter paper would be highly relevant, given its remarkable contribution to the primary care system for NBs, such as in the heel prick test, for example.

Brazil's National Neonatal Screening Program (PNTN, in the Portuguese acronym), known as the “Heel Prick Test”, was created and implemented by the Ministry of Health under Directive MG/MS No. 822/01, 26 and is aimed at the early detection of disorders and diseases in NBs to ensure the appropriate intervention and potentiation of treatment. Almost 3 million children are born each year in Brazil, and the coverage of NB screening varies according to the state. Nevertheless, in 2017 (the most recent data), the national coverage of the heel prick test reached 85.8%, demonstrating the strong support of this program by the population. 27

The clearly greater adherence to the heel prick test than to prenatal testing of the mother underscores the bias against the prenatal program. Hence, to compensate for this lack, the IgG avidity test on filter paper can be of great value as part of the methodological approaches carried out in the heel prick test.

Detection of low-avidity IgG as early as in the heel prick test of NBs may streamline the diagnosis of congenital infection. According to Fonseca et al., 23 NBs exposed to *T. gondii* show elevated serum IgM and IgG levels, and when their IgG shows low avidity, they exhibit more severe symptoms of congenital toxoplasmosis. The aforementioned authors observed that high IgG avidity in NBs probably indicates a lower risk of infection by *T. gondii* . 28

The 10 discordant samples reported in item 1 of the Results section can be explained by the 61.1 to 99.3% sensitivity rate of the ELISA test and by the timeframe of the 2 ^nd^ blood collection, which, in some cases, was 3 months. Despite this slight divergence, the use of serology on samples collected on filter paper has already been standardized and is widely used, and the technique is considered highly efficient and reproducible. 29 30 31 32

As for the 4 samples that presented low IgG antibody avidity described under item 2 of the Results section, and considering that IgG crosses the placental barrier, this antibody may have been passed on to the fetus during pregnancy. However, this does not diminish its relevance, since it may indicate primary maternal infection, with a considerable risk of vertical transmission. This situation may indicate a recent infection or even a current production of low avidity IgG by the NB, which in both cases is extremely important for the diagnosis and earliest possible treatment. 33 34

The fact that not all samples with high IgG concentrations were collected in duplicate was due to the difficulty in communicating with the parents of the infants and to their lack of interest in allowing a second blood collection, as the infants were apparently asymptomatic. It should be noted that the lack of communication is one of the major problems in the diagnosis of congenital toxoplasmosis, as NBs are often born asymptomatic and only present sequelae months or even years after their birth. 30 35 36 37

Our data reveal that screening infants with high IgG titers, allied to avidity testing, can contribute to the tracking and early diagnosis of postnatal toxoplasmosis.

## Conclusion

The IgG avidity test proved to be efficient because it enabled the detection of patients with low avidity in 2.4% of the analyzed samples, contributing to the early identification of congenital toxoplasmosis in 50% of these samples, subsequently confirmed by Western Blot tests. Screening for toxoplasmosis in NBs with high IgG titers, allied with avidity testing, can be performed on filter paper and easily included in the current heel prick test. Thus, it can contribute to tracking and early diagnosis, since congenital toxoplasmosis is difficult to diagnose and depends on several factors, particularly on those pertaining to the age of the fetus when infection set in, and on the absence of symptoms in infected infants, which makes their identification even more difficult. The data reported here indicate that the avidity test may be another valuable method for the diagnosis of congenital toxoplasmosis in NBs. This method is inexpensive and easy to implement on blood samples that present high concentrations of IgG detected in the heel prick test, the basic test of the postnatal program, offered nationwide by the SUS.
